# Clinical and etiological profile of epilepsy at the regional hospital center of Tahoua (Niger): A 4‐year retrospective study

**DOI:** 10.1002/brb3.2301

**Published:** 2021-07-21

**Authors:** Moussa Toudou‐Daouda, Abdoul Kadir Ibrahim‐Mamadou

**Affiliations:** ^1^ Department of Neurology National Hospital of Niamey Niamey Niger; ^2^ Department of Medicine and Medical Specialties Regional Hospital Center of Tahoua Tahoua Niger

**Keywords:** epilepsy, hospital‐based study, Niger, sub‐Saharan Africa, Tahoua

## Abstract

**Objective:**

We aimed to evaluate epilepsy management at the regional hospital center (RHC) of Tahoua (Niger) to determine the intrahospital deficiencies to optimize to improve the management of people with epilepsy.

**Methods:**

A descriptive retrospective study was carried out at the Psychiatric Unit of the RHC of Tahoua between January 1, 2016 and December 31, 2019. We collected from the registers of consultation all patients followed by nurse technicians in mental health for epilepsy whose diagnosis was made by nonspecialist physicians or internists. The study included patients with epilepsy who had a medical follow‐up at least 6 months.

**Results:**

Of the 2022 patients seen during the period of the study, 246 patients were consulted for epilepsy with a hospital frequency of 12.2%. The mean age was 22.38 years, with a slight predominance of men (57.7%). Only generalized tonic‐clonic seizures (95.1%) and focal‐aware seizures (4.9%) were reported. The main etiologies were cerebral malaria (18.7%), bacterial meningitis (8.1%), and head trauma (4.9%). In 60.2% of cases, the etiology was undetermined, but the etiological diagnosis investigation was incomplete (CT scan not done). Only the first‐generation antiepileptic drugs were used. Only 2.8% of the patients had drug‐resistant epilepsy, and 97.2% of the patients had controlled seizures.

**Conclusion:**

The study shows a predominance of infectious causes, particularly cerebral malaria. We found a high proportion of epilepsies with unknown etiologies with incomplete workup. The RHC of Tahoua should facilitate access to the CT scan for people with epilepsy to improve etiological diagnosis investigation.

## Abstract 2

In Niger, epilepsy remains a major public health concern. The high prevalence of infectious diseases in Niger plays a major role in the prevalence of epilepsy.

The hospital frequency of epilepsy at the regional hospital center of Tahoua is 12.2%.

Infectious causes (26.8%) and head injuries (4.9%) were the most common identified etiologies.

## 1 INTRODUCTION

Epilepsy is one of the most common chronic neurological diseases that affects more than 50 million people worldwide, and almost 80% of them live in developing countries (Ngugi et al., 2010). In developing countries, particularly in sub‐Saharan African countries such as Niger, epilepsy still constitutes a major public health concern where its prevalence is high, especially in rural areas and mainly among young people (Assadeck et al., 2019). This disease requires qualified care, given its high prevalence.

Until May 2018, the public health infrastructures of Niger consisted of 1057 integrated health centers, 2466 health huts, seven regional hospital centers, seven mother and child health centers, and four tertiary care hospitals with national referral maternity (OMS, 2018). Niger's health system also included 387 private establishments (such as 346 medical offices, 36 clinics, two nonprofit hospitals, and two private centers specializing in ophthalmology and trauma), 120 private pharmacies, 11 laboratories, and 19 private health schools. In Niger, people with epilepsy are mainly cared for by nonphysician health care workers, nonspecialist physicians, and non‐neurologist physicians because, until May 2018, the country had only seven neurologists who worked in the hospitals of Niamey (capital of Niger). Nonphysician health care workers are mainly represented by nurse technicians in mental health who are trained in the management of epilepsy and psychiatric diseases. In the Tahoua region (Niger), the majority of people with epilepsy are followed at the Psychiatric Unit of the Regional Hospital Center (RHC) of Tahoua by nurse technicians in mental health assisted by nonspecialist physicians and internists. In the case of diagnostic difficulty, the patient is referred to Niamey for neurological consultation, and for eventual additional examinations useful to make an etiological diagnosis. This demonstrates not only the unavailability of diagnostic tools such as electroencephalogram (EEG) and neuroimaging exams but also the lack of neurologists at the RHC of Tahoua.

In Niger, only one study was carried out on the clinical characteristics of epilepsy in a tertiary care referral center of Niamey (Assadeck et al., 2019). Thus, the present study was designed to evaluate epilepsy management at the RHC of Tahoua to determine the intrahospital deficiencies to optimize to improve the management of people with epilepsy.

## 2 MATERIALS AND METHODS

### 2.1 Study design

A descriptive retrospective study was carried out at the Psychiatric Unit of the RHC of Tahoua (Niger, a sub‐Saharan African country) between January 1, 2016 and December 31, 2019. The RHC of Tahoua is a major referral center for urban care in the Tahoua region, which covers an area of 75,000 m² and comprises eight buildings with 282 beds. To date, this hospital attracts people from all corners of the Tahoua region and those from surrounding areas to seek medical care in various areas of health. The Psychiatric Unit of this hospital has a small reception capacity (only 19 beds). Patients with mental disorders and epilepsy are mainly followed in consultation by nurse technicians in mental health assisted by nonspecialist physicians and internists. In Niger, nurse technicians in mental health are trained at the Faculty of Health Sciences of the Abdou Moumouni University of Niamey. The duration of their training is three years, and at the end of the training, they receive a license in mental health. Their role is to significantly assist psychiatrists and replace them in areas where there are no psychiatrists. During their training, nurse technicians in mental health receive training modules on psychiatric illnesses and epilepsy from the psychiatrists and neurologists of the Faculty of Health Sciences of Niamey. During their training, they carry out their practical training at the Department of Psychiatry and the Department of Emergency of the National Hospital of Niamey, where they learn the management of psychiatric conditions and epilepsy. After acquiring good experience in the management of psychiatric conditions and epilepsy, these nurse technicians in mental health are assigned in the integrated health centers, health districts, and regional hospital centers where they play the role of psychiatrists when these latter are not available.

### 2.2 Patients

The study included people of all ages who were followed at the Psychiatric Unit of the RHC of Tahoua for epilepsy by nurse technicians in mental health, whose diagnosis was made by nonspecialist physicians or internists. Once the diagnosis of epilepsy is made, antiepileptic treatment is initiated by nonspecialist physicians or internists. The study included patients with epilepsy who had a medical follow‐up at least 6 months.

The sociodemographic (age of diagnosis, gender, profession, provenance, marital status, and past medical history), clinical (age of first seizures, type of seizure, and associated clinical signs), etiological, and therapeutic data had been collected retrospectively from the registers of consultation during the first consultation for each patient. The outcomes data had been collected during the follow‐up visits. The patients were followed at a frequency of 3 months. For the profession, farmers, breeders, and butchers were considered as peasants. Brain imaging (computed tomography [CT] scan) and EEG were only performed in a few patients due to the unavailability of these examinations at the RHC of Tahoua. CT scan was done in some cases of suspected cerebral lesions in patients who had financial resources, and who had agreed to go to Niamey to get this examination done. Regarding the EEG, only patients who agreed to go to Niamey had benefited from this examination. The laboratory examinations available at the RHC of Tahoua were performed in some patients when it was necessary. During follow‐up visits, all patients were evaluated, and their antiepileptic treatment response was also evaluated. In case of persistence of epileptic seizures, the antiepileptic treatment was adapted by nonspecialist physicians or internists. Retrospectively, a patient was considered to have drug‐resistant epilepsy in case of failure to achieve sustained seizure freedom despite adequate trials of two well‐tolerated and appropriately chosen antiepileptic drugs (AEDs) prescribed as monotherapies or in combination. A patient was considered to have controlled seizures when the AED intake leads to a total seizures regression, and the patient no longer presents seizures.

### 2.3 Ethical approval

The local Ethics Committee of RHC of Tahoua (Niger) considered the study to be a service evaluation and ruled that no formal ethics approval was required for this study because patient management was not affected. The written informed consent was waived by the local Ethics Committee because of the retrospective and anonymous nature of the data.

### 2.4 Statistical analysis

In the descriptive analysis of the data, patient characteristics were expressed as percentages for the qualitative variables and mean ± SD for the quantitative variables. The chi‐square test of Pearson and Fisher's exact test were used to compare the proportions of the qualitative variables. *p*‐Values <.05 were considered statistically significant. All statistical analyses were performed using the IBM SPSS statistical software package version 22.0 (SPSS Inc.).

## 3 RESULTS

### 3.1 Sociodemographic characteristics

Of the 2022 patients seen at the Psychiatric Unit of the RHC of Tahoua between January 1, 2016 and December 31, 2019, 246 patients had epilepsy with a hospital frequency of 12.2%. Table 1 summarizes the sociodemographic characteristics of the patients. The study included 142 men (57,7%) and 104 women (42,3%) with a sex ratio of 1.36. One hundred and twenty‐five patients (50.2%) were from urban areas. The mean age of the patients was 22.38 ± 11.89 years (range: 3–65 years). Single and married patients were the most represented in 51.2% and 43.9% of cases, respectively. Peasant patients represented 46.3% of cases. The main etiological factors of the patients were cerebral malaria (13%), bacterial meningitis (6.5%), and drug addiction (4.9%).

**TABLE 1:** Sociodemographic characteristics of the patients (*n* = 246)

**Variables**	**Number (%)**
Sex, *n* (%)	
Males	142 (57.7)
Females	104 (42.3)
Sex ratio (male/female)	1.36
Age (year), *n* (%)	
Mean	22.38 ± 11.89
Range	3 and 65
0–4	7 (2.8)
5–9	30 (12.2)
10–14	36 (14.6)
15–17	27 (11)
18–27	87 (35.4)
28–37	35 (14.2)
38–47	15 (6.1)
48–59	5 (2)
≥60	4 (1.6)
Provenance, *n* (%)	
Rural environment	121 (49.2)
Urban environment	125 (50.8)
Profession, *n* (%)	
Students	16 (6.5)
Peasants	114 (46.3)
Doctors	1 (0.4)
No profession	115 (46.7)
Marital status, *n* (%)	
Married	108 (43.9)
Single	126 (51.2)
Divorced	4 (1.6)
Widows/widowers	8 (3.3)
Past medical history, *n* (%)	
Dystocic delivery	11 (4.5)
Cerebral malaria	32 (13)
Bacterial meningitis	16 (6.5)
Head injury	9 (3.7)
Cerebrovascular disease	4 (1.6)
Drug addiction	12 (4.9)
None	162 (65.9)

### 3.2 Clinical characteristics

The mean age of first seizures was 18.96 ± 13.36 years (range: 0 and 65 years) (Table 2). The age of first seizures was less than 10 years in 29.2% of the patients and less than 20 years in 59.7% of the patients. Generalized tonic‐clonic seizures (95.1%) and focal aware seizures (4.9%) were the two seizure types identified in this study. Loss of acquisitions or psychomotor decline was the most frequent associated clinical sign (6.5%), followed by the hemicorporeal motor deficit (1.6%).

**TABLE 2:** Clinical, etiological, and therapeutic characteristics as well as the outcomes during follow‐up visits of the patients (*n* = 246)

**Variables**	**Number (%)**
Seizures type, *n* (%)	
Generalized tonic‐clonic seizures	234 (95.1)
Focal aware seizures	12 (4.9)
Age of first seizures (year), *n* (%)	
Mean	18.96 ± 13.36
Range	0 and 65
<5	22 (8.9)
5–9	50 (20.3)
10–14	29 (11.8)
15–19	46 (18.7)
20–29	52 (21.1)
30–39	23 (9.3)
40–49	15 (6.1)
≥50	9 (3.7)
Associated clinical signs, *n* (%)	
Loss of acquisitions or psychomotor decline	16 (6.5)
Hemiparesis/hemiplegia	4 (1.6)
Language disorders	2 (0.8)
Etiologies and risk factors, *n* (%)	
Infectious causes	66 (26.8)
Cerebral malaria	46 (18.7)
Bacterial meningitis	20 (8.1)
Structural causes	23 (9.3)
Posttraumatic epilepsy	12 (4.9)
Poststroke epilepsy	4 (1.6)
Hypoxic‐ischemic encephalopathy due to birth asphyxia	7 (2.8)
Toxic causes	9 (3.7)
Unknown	148 (60.2)
Treatment, *n* (%)	
VPA	104 (42.3)
CBZ	37 (15)
PB	61 (24.8)
VPA–CBZ combination	8 (3.3)
PB–CBZ combination	36 (14.6)
Number of consultations, *n* (%)	
Mean	6.84 ± 4.69
Range	3 and 48
<5	68 (27.6)
5–9	80 (32.5)
≥10	24 (9.8)
Unspecified	74 (30.1)
Outcomes during follow‐up visits, *n* (%)	
Seizures control	239 (97.2)
Drug‐resistance	7 (2.8)

Abbreviations: CBZ, carbamazepine; PB, phenobarbital; VPA, sodium valproate.

Generalized tonic‐clonic seizures were significantly more frequent in patients aged 20–39 years (*p* = .001) (Table 3). We did not find a relationship between gender and seizures type.

**TABLE 3:** Sex and age groups by seizures type, etiologies, and treatments

**Variables**	**Total(*n* = 246)**	**Sex**			**Age groups (year)**				
		**Male(*n* = 142)**	**Female(*n* = 104)**	*p‐*Value	**<20(*n* = 105)**	**20–9(*n* = 117)**	**40–59(*n* = 20)**	**≥60(*n* = 4)**	***p‐*Value**
Seizures type, *n* (%)									
GTCS	234 (95.1)	135 (95.1)	99 (95.2)	.955*	101 (96.2)	114 (97.4))	18 (90)	1 (25)	.001#
FAS	12 (4.9)	7 (4.9)	5 (4.8)		4 (3.8)	3 (2.6)	2 (10)	3 (75)	
Etiologies, *n* (%)									
Cerebral malaria	46 (18.7)	26 (18.3)	20 (19.2)	.855*	35 (33.3)	11 (9.4)	0	0	<.001#
Bacterial meningitis	20 (8.1)	13 (9.2)	7 (6.7)	.492*	16 (15.2)	4 (3.4)	0	0	.001#
Posttraumatic epilepsy	12 (4.9)	11 (7.7)	1 (1)	.015#	2 (1.9)	10 (8.5)	0	0	.452#
Poststroke epilepsy	4 (1.6)	3 (2.1)	1 (1)	.482#	0	0	4 (20)	0	<.001#
HIEBA	7 (2.7)	3 (2.1)	4 (3.8)	.420#	5 (4.8)	2 (1.7)	0	0	.119#
Toxic causes	9 (3.7)	9 (6.3)	0	.009#	1 (1)	7 (6)	1 (5)	0	.166#
Unknown	148 (60.2)	77 (54.2)	71 (68.3)	.027*	46 (43.8)	83 (70.9)	15 (75)	4 (100)	<.001#
Treatment, *n* (%)									
VPA	105 (42.7)	59 (41.5)	46 (44.2)	.674*	42 (40)	54 (46.2)	8 (40)	1 (25)	.872#
CBZ	37 (15)	27 (19)	10 (9.6)	.042*	14 (13.3)	19 (16.2)	4 (20)	0	.683#
PB	61 (24.8)	29 (20.4)	32 (30.8)	.064*	24 (22.9)	30 (25.6)	7 (35)	0	.655#
VPA–CBZ combination	7 (2.8)	3 (2.1)	4 (3.8)	.420#	5 (4.8)	2 (1.7)	0	0	.119#
PB–CBZ combination	36 (14.6)	24 (16.9)	12 (11.5)	.241*	20 (19)	12 (10.3)	1 (5)	3 (75)	.651#

Abbreviations: CBZ, carbamazepine; FAS, focal aware seizures; GTCS, generalized tonic‐clonic seizures; HIEBA, hypoxic‐ischemic encephalopathy due to birth asphyxia; PB, phenobarbital; VPA, sodium valproate.

*p‐Value was calculated using the chi‐square test of Pearson.

#*p*‐Value was calculated using Fisher's exact test.

### 3.3 Etiological characteristics and risk factors

Cerebral malaria was the most common etiology (18.7%), followed by bacterial meningitis (8.1%) and head trauma (4.9%) (Table 2). In 60.2% of the patients, the etiology was undetermined, but the etiological diagnosis investigation was incomplete (cerebral CT scan not done).

Head injuries were significantly more frequent in men than in women (7.7% vs. 1%; *p =* .015) as well as toxic causes (6.3% vs. 0%; *p =* .009). Significantly, cerebral malaria (*p <* .001) and bacterial meningitis (*p =* .001) were more common in patients younger than 20 years. Poststroke epilepsies were significantly more frequent in patients older than 40 years (*p <* .001). Cerebral malaria was more frequent in patients living in urban areas than those living in rural areas (26.4% vs. 10.7%; *p =* .002) (Table 4). Toxic causes were more frequent in peasant patients (*p =* .014).

**TABLE 4:** provenance and profession by seizures type, etiologies, and treatments

**Variables**	**Total(*n* = 246)**	**Provenance**			**Profession**				
		**Urban(*n* = 125)**	**Rural(*n* = 121)**	***p‐*Value**	**Students(*n* = 16)**	**Peasants(*n* = 114)**	**Doctors(*n* = 1)**	**None(*n* = 115)**	***p‐*Value**
Seizures type, *n* (%)									
GTCS	234 (95.1)	121 (96.8)	113 (93.4)	.248#	16 (100)	108 (94.7)	1 (100)	109 (94.8)	.680#
FAS	12 (4.9)	4 (3.2)	8 (6.6)		0	6 (5.3)	0	6 (5.2)	
Etiologies, *n* (%)									
Cerebral malaria	46 (18.7)	33 (26.4)	13 (10.7)	.002*	7 (43.8)	13 (11.4)	0	26 (22.6)	.470#
Bacterial meningitis	20 (8.1)	12 (9.6)	8 (6.6)	.391*	3 (18.8)	6 (5.3)	0	11 (9.6)	.774#
Posttraumatic epilepsy	12 (4.9)	5 (4)	7 (5.8)	.516*	0	9 (7.9)	0	3 (2.6)	.221#
Poststroke epilepsy	4 (1.6)	3 (2.4)	1 (0.8)	.622#	0	3 (2.6)	0	1 (0.9)	.488#
HIEBA	7 (2.7)	5 (4)	2 (1.7)	.447#	1 (6.2)	0	0	6 (5.2)	.085#
Toxic causes	9 (3.7)	4 (3.2)	5 (4.1)	.746#	0	9 (7.9)	0	0	.014#
Unknown	148 (60.2)	63 (50.4)	85 (70.2)	.001*	5 (31.2)	74 (64.9)	1 (100)	68 (59.1)	.751#
Treatment, *n* (%)									
VPA	105 (42.7)	56 (44.8)	49 (40.5)	.496*	6 (37.5)	50 (43.9)	1 (100)	48 (41.7)	.927#
CBZ	37 (15)	17 (13.6)	20 (16.5)	.521*	2 (12.5)	24 (21.1)	0	11 (9.6)	.043#
PB	61 (24.8)	26 (20.8)	35 (28.9)	.141*	4 (25)	25 (21.9)	0	32 (27.8)	.363#
VPA–CBZ combination	7 (2.8)	5 (4)	2 (1.7)	.269#	1 (6.2)	1 (0.9)	0	5 (4.3)	.309#
PB–CBZ combination	36 (14.6)	21 (16.8)	15 (12.4)	.330*	3 (18.8)	14 (12.3)	0	19 (16.5)	.557#

*Note*: Peasants: The farmers, breeders, and butchers were considered peasants.

Abbreviations: CBZ, carbamazepine; FAS, focal aware seizures; GTCS, generalized tonic‐clonic seizures; HIEBA, hypoxic‐ischemic encephalopathy due to birth asphyxia; PB, phenobarbital; VPA, sodium valproate.

*p‐Value was calculated using the chi‐square test of Pearson.

#*p*‐Value was calculated using Fisher's exact test.

### 3.4 Therapeutic characteristics

Only the first‐generation AEDs (sodium valproate [VPA], carbamazepine [CBZ], and phenobarbital [PB]) were prescribed in this study. VPA was the most used AED (42.3%). The CBZ–PB combination was the most used (14.6% of all cases).

Significantly, CBZ was more prescribed in men than in women (19% vs. 9.6%; *p* ***=*** .042).

### 3.5 Outcomes during follow‐up visits

The patients were followed at a frequency of 3 months. The mean number of follow‐up visits was 6.84 ± 4.69 (range: 3 and 48). The number of follow‐up visits was ≥5 in 42.3% of the patients. In 97.2% of the patients, the seizures had been controlled. The drug resistance rate was 2.8% of cases.

Significantly, we found an association between drug resistance and bacterial meningitis (*p <* .001) and hypoxic‐ischemic encephalopathy due to birth asphyxia (*p <* .001) (Table 5). Patients with drug‐resistant epilepsy were significantly on dual therapy (*p <* .001).

**TABLE 5:** Outcomes during follow‐up visits by sex, age groups, profession, provenance, etiologies, treatments, and number of consultations

		**Outcomes during follow‐up visits**		
**Variables**	**Total (*n* = 246)**	**Seizures control (*n* = 239)**	**Drug‐resistance (*n* = 7)**	***p‐*Value**
**Sex, *n* (%)**				
Male	142 (57.7)	136 (56.9)	6 (85.7)	.129
Female	104 (42.3)	103 (43.1)	1 (14.3)	
Age groups (year), *n* (%)				
<20	105 (42.7)	100 (41.8)	5 (71.4)	.120
20–39	117 (47.6)	115 (48.1)	2 (28.6)	.308
40–59	20 (8.1)	20 (8.4)	0	.426
≥60	4 (1.6)	4 (1.7)	0	.731
Provenance, *n* (%)				
Rural environment	121 (49.2)	119 (49.8)	6 (85.7)	.061
Urban environment	125 (50.8)	120 (50.2)	1 (14.3)	
Profession, *n* (%)				
Students	16 (6.5)	15 (6.3)	1 (14.3)	.398
Peasants	114 (46.3)	113 (47.3)	1 (14.3)	.085
Doctors	1 (0.4)	1 (0.4)	0	.864
No profession	115 (46.7)	110 (46)	5 (71.4)	.185
Etiologies, *n* (%)				
Cerebral malaria	46 (18.7)	46 (19.2)	0	.199
Bacterial meningitis	20 (8.1)	16 (6.7)	4 (57.1)	<.001
Posttraumatic epilepsy	12 (4.9)	11 (4.6)	1 (14.3)	.242
Poststroke epilepsy	4 (1.6)	4 (1.7)	0	.731
HIEBA	7 (2.7)	5 (2.1)	2 (28.6)	<.001
Toxic causes	9 (3.7)	9 (3.8)	0	.602
Unknown	148 (60.2)	148 (61.9)	0	.001
Treatment, *n* (%)				
VPA	105 (42.7)	104 (43.5)	0	.022
CBZ	37 (15)	37 (15.5)	0	.260
PB	61 (24.8)	61 (25.5)	0	.124
VPA–CBZ combination	7 (2.8)	5 (2.1)	3 (42.9)	<.001
PB–CBZ combination	36 (14.6)	32 (13.4)	4 (57.1)	.001
Number of consultations, *n* (%)				
<5	68 (27.6)	68 (28.4)	0	.098
5–9	80 (32.5)	76 (31.8)	4 (57.1)	.159
≥10	24 (9.8)	23 (9.6)	1 (14.3)	.683
Unspecified	74 (30.1)	72 (30.1)	2 (28.6)	.930

*Note*: Peasants: The farmers, breeders, and butchers were considered as peasants.

Abbreviations: CBZ, carbamazepine; HIEBA, hypoxic‐ischemic encephalopathy due to birth asphyxia; PB, phenobarbital; VPA, sodium valproate.

*p*‐Value was calculated using Fisher's exact test.

## 4 DISCUSSION

The present study shows a hospital frequency of epilepsy of 12.2% at the Psychiatric Unit of the RHC of Tahoua. A study conducted at the tertiary level at the National Hospital of Niamey (Assadeck et al., 2019) reported a higher hospital frequency (29.5%). This higher hospital frequency of epilepsy at the National Hospital of Niamey could be explained by the fact that this hospital is one of the largest referral centers of the country in the management of neurological conditions. On the other hand, the low hospital frequency of epilepsy in the RHC of Tahoua could be explained by the fact that epilepsy is considered by many people as a demon possession and that the treatment is based on traditional medicine. For example, in a cross‐sectional survey about epilepsy knowledge of primary and secondary school teachers in the city of Tahoua who are considered as intellectuals (Toudou‐Daouda & Ibrahim‐Mamadou, 2020), 16.2% of the respondents considered epilepsy as a demon possession and 13.% of them think that the treatment is based on traditional medicine.

In the present study, the mean age of the patients was 22.38 years, with a slight predominance of male sex (sex ratio 1.36). This predominance of the male sex has been reported in several African sub‐Saharan studies (Houinato et al., 2013; Igwe et al., 2014; Ogunrin et al., 2014) and recently the study carried out in Niger at the National Hospital of Niamey (Assadeck et al., 2019). On the other hand, a predominance of the female sex has been reported in other studies (Osuntokun et al., 1987, 1982; Rwiza et al., 1992; Simms et al., 2008). The male overrepresentation in the majority of studies could be explained by the fact that the disease is underreported among young women of marriageable age in sub‐Saharan Africa. The declaration of this disease in a young woman could condemn her to spend her whole life being single because men will not want her in marriage because of their religious beliefs. Thus, parents hide the illness of their daughters to protect them and avoid sociocultural rejection. In the cross‐sectional survey about epilepsy knowledge of primary and secondary school teachers in the city of Tahoua, 41.9% of the participants considered epilepsy as a contagious disease, and 73.2% of them would not allow their children to marry someone with epilepsy (Toudou‐Daouda & Ibrahim‐Mamadou, 2020).

The mean age of first seizures was 18.96 years in the present study. The age of first seizures was less than 10 years in 29.2% of the patients and less than 20 years in 59.7% of the patients. Higher proportions for an age of first seizure less than 20 years have been reported in Burkina Faso (Nitiéma et al., 2012; [65.8%]) and Kenya (Munyoki et al., 2010; [77.8%]). On the other hand, a lower proportion than our findings for an age of first seizures less than 10 years has been reported in Cameroon (Kamgno et al., 2003; [24.6%]).

As in the present study, many studies from sub‐Saharan Africa showed a predominance of generalized tonic‐clonic seizures (Assadeck et al., 2019; Igwe et al., 2014; Kamgno et al., 2003; Mmbando et al., 2018). However, a predominance of focal‐onset seizures has been reported in other studies in sub‐Saharan Africa (Newton & Gero, 1984; Osuntokun et al., 1987; Van Der Waals et al., 1983). The report of only two seizure types in the present study could be explained by the fact that epilepsy management had been provided by health workers not well experienced in the management of this disease. Certain types of focal to bilateral tonic‐clonic seizures tend to be considered as generalized seizures.

In the present study, an etiology has been identified in 39.8% of the patients. Infectious causes were the most frequent (26.8%), followed by the structural causes (9.3%). Cerebral malaria was the most common infectious cause (18.7%), followed by bacterial meningitis (8.1%). The most common structural causes were head trauma (4.9%) and hypoxic‐ischemic encephalopathy due to birth asphyxia (2.8%). In the study carried out in Niamey at the National Hospital of Niamey, the structural causes were the most frequent dominated by cerebrovascular disease and head injuries (Assadeck et al., 2019). Two Nigerian studies reported a predominance of head injuries and febrile convulsions in childhood as the main etiological factors (Ogunrin et al., 2014, 1987). As in the present study, the predominance of infectious causes has been reported in a study from Togo (Balogou et al., 2007). In the present study, the predominance of cerebral malaria in patients living in urban areas than those living in rural areas could be explained by the fact that in Niger, there are more stagnant waters in cities than in the countryside. These stagnant waters are a source of reproduction for mosquitoes, which are the vector agents of malaria.

In the present study, only the first‐generation AEDs (VPA, CBZ, and PB) were prescribed. A Nigerian study also reported the prescription of only the first‐generation AEDs (Igwe et al., 2014). As in the study carried out at the National Hospital of Niamey (Assadeck et al., 2019), VPA was the most AED used in the present study. In Mali, a study reported phenytoin as the most AED used (Nimaga et al., 2002). In the present study, newer generation AEDs were not used because they are expensive for patients who are farmers in the majority of cases and have limited access to these drugs. Although first‐generation AEDs are not expensive, the rupture of these treatments in pharmacies is a recurrent problem in Niger, exposing patients to the risk of epileptic seizures due to drug withdrawal. However, this notion of the rupture of AEDs was not reported in the present study. Possibly, this information was neglected and not noted in the registers of consultation by nurse technicians in mental health during follow‐up visits. We found only 2.8% of cases of drug‐resistance in the present study. In the literature, it is estimated that 30%‐‐40% the of patients with epilepsy will develop drug‐resistant epilepsy (Kalilani et al., 2018). Our results are very far from the data in the literature. This low rate of drug‐resistance in the study presence could be explained by (1) medical follow‐up provided by nurse technicians in mental health (donation of AEDs and search for epileptic seizures) who do not recognize certain types of focal onset seizures. Besides, nonspecialist physicians and internists are called upon by nurse technicians in mental health in cases of persistent trues generalized tonic‐clonic seizures or focal onset seizures, and (2) the absence of electroencephalographic monitoring responsible for the failure to detect certain types of focal onset seizures not recognized by nurse technicians in mental health during medical follow‐up.

### 4.1 Limitations of the study

The first limitation of the study is sample size. Second, the unavailability of complementary examinations for an etiological investigation of epilepsy at the RHC of Tahoua such as neuroimaging exams. This explains the high proportion of patients with epilepsy without a defined etiology. Third, the retrospective nature of this study explains why some details were not provided especially seizure frequency in patients with drug‐resistant epilepsy. Fourth, the low prevalence of drug resistance suggests that certain types of focal‐onset seizures are unrecognized because the medical follow‐up was provided essentially by nurse technicians in mental health.

## 5 CONCLUSIONS

Our study shows a predominance of infectious causes (26.8%), certainly due to a high prevalence of infectious diseases in Niger, such as malaria and meningitis. Also, the study found only 2.8% of drug resistance, which is far and away very low compared to the literature data. On the other hand, the present study shows limited access to diagnostic tests of epilepsy at the RHC of Tahoua, such as the CT scan explaining why 60.2% of the patients did not have an identified etiology. To improve the quality of epilepsy management at the RHC of Tahoua, it is necessary to facilitate for people with epilepsy the accessibility to the cerebral imaging such as CT scan. It is also necessary to organize continuous training sessions for the health workers who are already in their workstation (nurse technicians in mental health, nonspecialist physicians, and internists) to improve their knowledge of epilepsy management. Finally, young people must be encouraged to specialize in neurology, and the fight against infectious diseases must be well undertaken.

## CONFLICT OF INTEREST

The authors declare no conflict of interest.

## AUTHORS CONTRIBUTION

**Abdoul Kadir Ibrahim‐Mamadou** substantially contributed to the conception, drafting, design of the work, the acquisition, analysis, and interpretation of data for the work, and **Moussa Toudou‐Daouda** contributed to interpretation of data for the work, drafting the work and revising it critically for important intellectual content. The authors read and approved the final submitted version of the manuscript.

### PEER REVIEW

The peer review history for this article is available at https://publons.com/publon/10.1002/brb3.2301.

## Data Availability

All data generated or analyzed during this study are included in this published article.
